# Reciprocal Effects of Litter from Exotic and Congeneric Native Plant Species via Soil Nutrients

**DOI:** 10.1371/journal.pone.0031596

**Published:** 2012-02-16

**Authors:** Annelein Meisner, Wietse de Boer, Johannes H. C. Cornelissen, Wim H. van der Putten

**Affiliations:** 1 Department of Terrestrial Ecology, Netherlands Institute of Ecology (NIOO-KNAW), Wageningen, The Netherlands; 2 Department of Microbial Ecology, Netherlands Institute of Ecology (NIOO-KNAW), Wageningen, The Netherlands; 3 Systems Ecology, Department of Ecological Science, Faculty of Earth and Life Sciences, Vrije Universiteit (VU) Amsterdam, Amsterdam, The Netherlands; 4 Laboratory of Nematology, Wageningen University, Wageningen, The Netherlands; Duke University, United States of America

## Abstract

Invasive exotic plant species are often expected to benefit exclusively from legacy effects of their litter inputs on soil processes and nutrient availability. However, there are relatively few experimental tests determining how litter of exotic plants affects their own growth conditions compared to congeneric native plant species. Here, we test how the legacy of litter from three exotic plant species affects their own performance in comparison to their congeneric natives that co-occur in the invaded habitat. We also analyzed litter effects on soil processes. In all three comparisons, soil with litter from exotic plant species had the highest respiration rates. In two out of the three exotic-native species comparisons, soil with litter from exotic plant species had higher inorganic nitrogen concentrations than their native congener, which was likely due to higher initial litter quality of the exotics. When litter from an exotic plant species had a positive effect on itself, it also had a positive effect on its native congener. We conclude that exotic plant species develop a legacy effect in soil from the invaded range through their litter inputs. This litter legacy effect results in altered soil processes that can promote both the exotic plant species and their native congener.

## Introduction

Plant species can be introduced into new ecosystems by humans via transport, tourism, trade [1], [2] or changes in climate [3], [4], [5]. Some of these introductions result in biological invasions, which can have profound effects on the invaded habitats and the biodiversity therein [6], [7]. One of the strongest impacts of exotic plant species on ecosystem processes operates via altered quality of litter inputs, which can alter the cycling of nutrients [8], [9], [10]. These altered soil processes have been hypothesized to provide a positive feedback to the exotic plant species through changes in litter inputs [9], [11], [12], [13], but there are very few experimental tests showing that exotic plants indeed influence the legacy of the soil to their own benefit [10]. Here, we present results of an experimental study on litter effects of exotic and congeneric plant species, which are native in the invaded habitat, on soil processes and individual performance of exotic and native congener.

Differences in initial litter chemistry between exotic and native plant species are important for soil processes involved in litter decomposition [14], [15] and are mediated indirectly by the soil decomposer subsystem [16], [17], [18]. For example, a higher lignin content can slow down the phased processes of litter breakdown [19], because this recalcitrant component needs specialist lignolytic fungi for degradation and can shield the more easily available components (*e.g*. cellulose) from decomposers during the earliest phases of litter breakdown [20], [21]. Therefore, litter inputs of exotic plant species that differ in litter quality from native species have been shown to increase or decrease soil processes [22], [23], [24], which may remain in the soil as a legacy.

These litter legacies can affect the performance of exotic or native plant species [25], [26]. When litter deposition increases the soil nutrient status, this may create a positive legacy effect to the subsequent plant species, either native or exotic (Fig. 3.11c in [27]). For example, litter addition from an exotic grass has been observed to increase biomass of the exotic grass itself and of a native shrub [28]. In contrast, litter can create a negative legacy effect when litter releases compounds into the soil during litter decomposition that inhibit plant growth [29], [30]. A variety of long-term soil legacy effects of exotic plant species has been reported, including positive as well as negative legacy effects to native plant species [31], [32].

Altered cycling of nutrients by exotic plant species is often hypothesized to promote exotic plant species exclusively (e.g. [33], [34], [35]). A relatively large number of studies have analyzed exotic litter effects in a context of plant community interactions. However, less is known about individual effects of exotic plant litter on exotic and native plant species [10]. Here, we study if the legacy of litter from exotics and congeneric natives reciprocally affect their performance when grown in monocultures via changes in soil processes. When litter of exotic plant species is of higher quality than of native plant species, this may increase soil nutrient mineralization [33], [36] and nutrient availability [37], [38]. Recently established exotic plant species in the Netherlands may have higher litter quality than congeneric native species [39]. Therefore, we test the hypothesis that litter from these exotic plant species provides a positive feedback to itself and inhibits natives through soil legacy effects. In order to avoid confounding effects due to major differences in plant chemistry and other traits that might differ between species [40], we compared exotic plant species with congeneric natives that co-occur in the invaded habitat.

Our hypothesis was tested by three experiments. In the first two experiments, we tested how soil mixed with litter from exotic plant species influenced soil respiration, soil mineralization and soil availability of nitrogen compared to soil mixed with litter from native plants species. In the third experiment, we tested how decomposing litter from exotic and native plant species affected germination rates and plant biomass of both exotic and native plant species. We performed the experiments with three genera of exotic and congeneric native plants that all co-occur in the same invaded habitat (Table 1).

**Table 1:** Plant species used in experiments.

Plant name^1^	Plant origin^2^	Time of introduction^2^	Litter chemistry		
			% C	% N	Lignin (mg C/g litter)
*Artemisia biennis*	North-Asia	1950–1975	44	2.5	121
*Artemisia vulgaris*	Native^3^		46	1.7	205
*Rorippa austriaca*	East Europe	1900–1925	35	1.3	43
*Rorippa sylvestris*	Native^3^		39	2.2	84
*Senecio inaequidens*	South-Africa	1925–1950	46	2.3	113
*Senecio jacobaea* 4	Native^3^		44	1.8	130

1 Nomenclature according to Van der Meijden [80].

2 [69].

3 Native to the Netherlands.

4 recently *Senecio jacobaea* has been renamed as *Jacobaea vulgaris* [81].

## Results

### Experiment 1: Soil respiration

**Figure 1:**
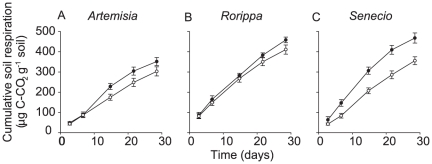
Mean cumulative soil respiration. (± SE). Measured in flasks with litter from exotic (filled circles) and native plant species (open circles) for *Artemisia* (a), *Rorippa* (b), and *Senecio* (c).

Exotic litter-inoculated soils showed (or in the case of *Rorippa* tended to show) a larger increase in cumulative respiration over time (Figure 1) as indicated by the Time by Origin interactions (Table 2).

**Table 2:** Repeated-measure ANOVA for soil respiration.

Factors	Plant genera								
	*Artemisia*			*Rorippa*			*Senecio*		
	d.f.	F	P	d.f.	F	P	d.f.	F	P
Between subject									
Origin (O)	1	2.77	0.13	1	0.96	0.36	1	13.9	0.004
Error	10			8			10		
Within subject									
Time (T)	1.4	361	<0.001	1.2	1141	<0.001	1.6	635	<0.001
T×O	1.4	5.47	0.027	1.2	4.50	0.054	1.6	13.9	<0.001
Error	14			9.9			16		

Litter from exotic versus native plant species (named Origin) of three genera (*Artemisia, Rorippa* and *Senecio*) were compared.

### Experiment 2: litter effects on soil N, enzyme activities and fungal biomass

**Figure 2:**
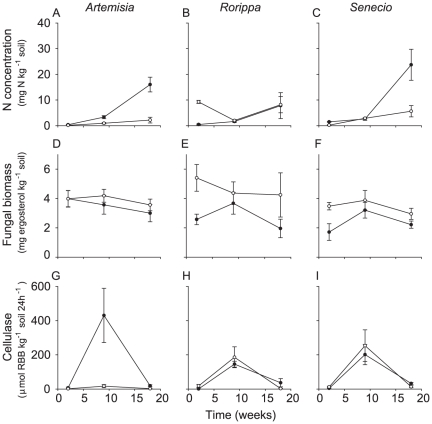
Effects of litter on nitrogen, fungal biomass and cellulase activity. Soil available inorganic nitrogen (N) (A, B, C), fungal biomass (D, E, F) and cellulase activity (G, H, I) in soil mixed with litter from exotic plant species (filled circles) and litter from native plant species (open circles). Means (± SE) are presented for *Artemisia* (A, D, G), *Rorippa* (B, E, H) and *Senecio* (C, F, I).

Soil with litter from exotic *Artemisia* and *Senecio* accumulated more inorganic N than soil with litter from their congeneric native species (Figure 2A and 2C), as indicated by the origin by time interaction (Table 3). There was also an origin by time interaction for *Rorippa* (Table 2), because soil with litter from exotic *R. austriaca* had lower N concentration than soil with litter from native *R. sylvestris* only after 2 weeks of incubation (Figure 2B). These differences in inorganic N accumulation between soils with litter from exotic and native plant species corresponds with the initial litter N concentrations (Table 1). Soil with litter from exotic plant species had less fungal biomass than soil with litter from native plant species in the case of *Rorippa* and *Senecio*, but not in the case of *Artemisia* (Table 3, Figure 2D, E, F). The highest activity of cellulase was observed after 9 weeks of incubation (Figure 2G, H, I, Table 3). Significant differences at peak activity were observed in the case of *Artemisia* (Table 3), where litter from exotic *A. biennis* induced the highest cellulase activity (Figure 2G). Mn-peroxidase activity in soil with litter was relatively low and did not show significant differences between soil with litter from exotics and natives (Table 3, see Figure S1A, B, C). Soil pH showed some significant, but minor differences (Table 3, see Figure S1D, E, F).

**Table 3:** ANOVA for effects of litter on soil properties.

Factors	Plant genera					
	*Artemisia* 1		*Rorippa* 1		*Senecio* 1	
	F	P	F	P	F	P
Soil Inorganic N						
Origin (O)	51.7	<0.001	12.0	0.005	18.6	<0.001
Time (T)	55.0	<0.001	6.82	0.01	34.7	<0.001
OxT	13.1	<0.001	10.6	0.002	5.91	0.008
Fungal biomass						
Origin (O)	0.80	0.38	5.10	0.043	7.57	0.01
Time (T)	1.00	0.38	0.49	0.63	2.70	0.087
OxT	0.20	0.82	0.54	0.59	0.85	0.44
Cellulase activity						
Origin (O)	28.1	<0.001	0.02	0.89	0.05	0.83
Time (T)	16.7	<0.001	16.5	<0.001	30.7	<0.001
OxT	2.97	0.07	5.77	0.018	3.03	0.07
Mn-peroxidase activity						
Origin (O)	0.89	0.35	0.44	0.42	0.18	0.67
Time (T)	14.2	<0.001	0.36	0.67	6.29	0.006
OxT	0.57	0.57	0.44	0.34	0.29	0.75
pH						
Origin (O)	4.40	0.046	11.9	0.005	4.00	0.057
Time (T)	43.9	<0.001	23.7	<0.001	36.8	<0.001
OxT	1.90	0.17	0.78	0.78	4.30	0.026

Litter from exotic or native species (Origin) were compared for three plant genera (*Artemisia*, *Rorippa* and *Senecio*) at three destructive sampling points (Time).

1 Numerator d.f. is 2 for time, 1 for origin and 2 for Time×Origin. Denominator d.f. is 24 for *Artemisia* and *Senecio* and 12 for *Rorippa* pair.

### Experiment 3: Litter effects on seedling germination and plant biomass

**Figure 3:**
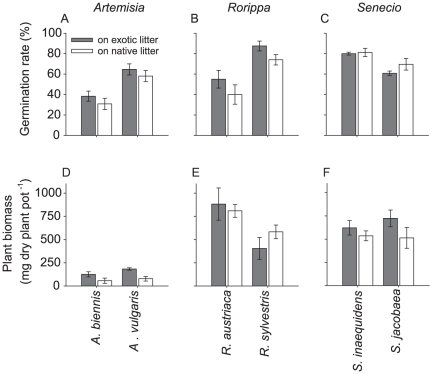
Effects of litter on germination rates and plant biomass production. Mean (± SE) for germination or (in the case of *Rorippa*) sprouting rate (A, B, C) and plant biomass (D, E, F) production of exotic and native plant species in litter from exotic (grey bars) or native plant species (white bars) belonging to three genera. Exotic plant species are: *A. biennis, R. austriaca* and *S. inaequidens*. Native plant species are: *A. vulgaris, R. sylvestris* and *S. jacobaea*. Significances of litter effects and plant effects are given in Table 4.

Seed germination and root sprouting of natives were not inhibited by litter from their congeneric exotic. In contrary, we observed a positive trend that litter from the exotic *R. austriaca* increased the rate of sprouting of both *R. sylvestris* and *R. austriaca* (Table 4, Figure 3). The rates of germination (and sprouting) of exotic plant species were lower than of natives for *Artemisia* and *Rorippa*, whereas the reverse was observed for *Senecio* (Figure 3A, B, C, Table 4).

**Table 4:** ANOVA for effects of litter effects on plant performance.

Factors	Plant genera					
	*Artemisia* 1		*Rorippa* 1		*Senecio* 1	
	F	P	F	P	F	P
Germination/sprouting						
Litter (L)	1.78	0.20	4.13	0.06	1.86	0.19
Plant (P)	23.7	<0.001	17.7	<0.001	13.9	0.002
LxP	0.02	0.88	0.06	0.81	0.79	0.39
Plant biomass						
Litter (L)	9.54	0.007	1.23	0.29	3.56	0.078
Plant (P)	1.04	0.32	7.47	0.016	0.03	0.87
LxP	0.02	0.89	1.52	0.24	0.86	0.37

Litter effects from exotic versus native plant species (Litter) on germination or (in the case of *Rorippa*) sprouting rates and plant biomass production as well as the differences between exotic and native plant species (Plant) within three genera (*Artemisia*, *Rorippa*, and *Senecio*).

1 Numerator d.f. is 1 for all factors. Denominator d.f. is 16, except for Rorippa-pair where denominator d.f. is 14.

Litter from exotics did not reduce biomass production of congeneric natives (Figure 3). Instead, *A. biennis and A. vulgaris* produced more biomass in soil with litter from the exotic *A. biennis* than from the native *A. vulgaris* (Table 4, Figure 3D). There was a similar trend for *Senecio* (Table 4, Figure 3F). *Rorippa austriaca* produced more biomass than *R. sylvestris*,whereas biomass was not different between exotic and native species in the case of *Artemisia* and *Senecio* (Table 4, Figure 3).

## Discussion

Our results reject the hypothesis that litter from exotic plant species inhibits native plant species while promoting themselves. Instead, we observed that if litter from an exotic plant species increased its own biomass production or germination rate, this litter also promoted biomass and germination of its native congener. Moreover, negative litter effects by litter from exotic plant species were not observed in our study. Our comparison was made within plant genera, but our results are in agreement with two other studies on litter effects of exotic species on natives. *Senecio jacobaea*, an exotic species introduced in New Zealand, increased biomass production of native plant species from New Zealand [41]. In addition, litter of an exotic grass in the USA favored not only its own biomass production, but also biomass production of a native shrub [28]. These studies and our results suggest that not only exotic plant species exclusively, but also native plant species may benefit from the litter of exotic plant species.

The positive effect of litter from exotic plant species may have been due to differences in initial litter quality, because litter from exotics contained less lignin and lower lignin: N ratios than litter of the congeneric natives (Table 1). The higher litter quality of exotic species may have increased microbial activity as shown by higher cumulative respiration rates, because the degradable carbon pool in litter from exotics was likely better accessible to decomposers than in litter from natives [42]. Based on cellulase-activities it seems that cellulose was only more available in litter from the exotic *A. biennis*. Soil available N concentrations reflected initial litter N concentrations, which were highest in litter from exotic *Artemisia* and *Senecio* species. In the case of *Rorippa*, there was no such an effect. The increased cumulative respiration rates and mineral N concentration in soil incubated with litter from exotic plant species could be the result of degradation of litter itself as well as from stimulation of degradation of soil organic matter (priming) [43]. This priming-induced increase of soil organic matter mineralization has also been proposed to be an important consequence of exotic grass invasion into hardwood forest [44]. Fungal biomass was more often lower in soil with litter from exotics than litter from natives, which is likely due to the lower initial lignin concentration of exotics [21], [45]. Therefore, litter from exotic species may change the soil food-web to a more bacterial dominated one if this litter is of higher quality than litter from native plant species [46], [47].

Other studies showed that differences in litter decomposition rates between exotic and native plant species strongly depend on initial litter quality (e.g. [23], [33], but see [48]). Our results indicate that these differences in litter decomposition rates between exotic and native plant species can result in altered soil processes and nutrient availability. Moreover, differences in initial litter quality between native and exotic plant species may explain the site-dependent differences in nutrient concentrations, litter decomposition and carbon mineralization between invaded and uninvaded sites in Europe [49], [50], [51].

The native plant species used in our study are also invasive in other parts of the world. It has been proposed that comparisons between exotic plant species and native plant species that are invasive elsewhere, may be complicated, as the natives have traits that can promote their invasiveness [52]. In that case, a congeneric comparison of exotics and natives should not result in differences, whereas our study showed that litter from exotics clearly promoted soil respiration and nitrogen availability compared with litter from natives. Species that are introduced into other regions often pass through environmental filters, which can result in rapid evolution of these plant species [53], [54]. As a result, invasive and native populations of the same species do not necessarily have the same traits [55], [56]. Our congeneric comparisons made it less likely that differences in litter effect may be due to secondary defense compounds exclusively produced by exotic plants [57]. Nevertheless, in cases of differences in secondary defense compounds, or when slow growing native plant species with poor litter quality are being replaced by fast growing exotics with high litter quality [58], it is possible that exotic species benefit disproportionally from their own litter.

Litter legacy effects are important for the dominance of individual plant species in plant communities in the next growing season [25], [26]. Litter legacies that increase soil nutrient concentrations may increase the dominance of exotic plant species when they take more advantage of these nutrients than the competing natives. Therefore, interactions with other mechanisms that increase the performance of exotics more than natives should be considered when explaining exotic plant dominance in ecosystems [59], [60]. For example, a modeling study showed that an exotic invasive wetland plant has likely evolved a mechanism to produce litter of lower quality that decomposes slower, which reduces the dominance of the native plant species due to competition for light [61]. Another mechanism that could interact with a positive litter legacy effect on soil processes is the release from belowground enemies when an exotic plant species invades a new range (e.g. [62], [63], [64]). Indeed, two exotics in our study have been shown to experience a less negative effect from their rhizosphere biota [65]. In that case, litter of exotic plants may cause a legacy effect favoring the exotic over natives when they are released from soil-borne enemies. Therefore, future experiments may be needed to untangle these interacting mechanisms, for example by growing exotic and native species in competition.

We conclude that monocultures of the exotic plant species and their congeneric native can benefit from increased soil nutrient availability through the legacy of exotic litter. Litter legacy effects on soil processes alone may, therefore, disproportionally benefit exotic over native plant species only in interaction with other mechanisms [66].

## Materials and Methods

### Ethics Statement

All necessary permits to collect soil and plant material from the Gelderse Poort region were obtained from Staatsbosbeheer regio Oost, the Netherlands.

### Plant selection

We made a phylogenetically controlled comparison of exotics and congeneric natives (e.g. [23], [37], [67]), to ensure that differences in litter effects would not be influenced by differences in major classes of plant chemistry within a plant pair. The three plant pairs all co-occurred in the same riverine habitat and the exotic and native congeners occurred in mixed stands [68]. Therefore, species interactions through litter are realistically occurring in the field. Three exotic and their congeneric native plant species were selected using the national standard list of the Dutch flora [39], [65], [69]. We chose exotic plant species that are recent invaders and have increased in frequency in the second half of the 20^th^ century in order to include exotic species with invasive potential [5]. Finally, a practical point was that sufficient amounts of litter, and seeds or root fragments had to be available to conduct the experiment. All plants co-occurred in the Gelderse Poort region, which is where the River Rhine enters the Netherlands. Three species pairs that could be selected according to the abovementioned criteria were: *Artemisia biennis* and *A. vulgaris*; *Rorippa austriaca* and *R. sylvestris*; *Senecio inaequidens* and *S. jacobaea* (Table 1). The three native species are all invasive in other parts of the world [70], [71], [72].

### Collection of plant and soil material

Soil, litter, seeds and root fragments were all collected from the Gelderse Poort region. Root fragments were collected for *Rorippa*, because this genus and especially the exotics has very difficult seeds to collect [73]. Soil was collected from 5 locations in Millingerwaard, a nature reserve within this region (51°52′N; 5°59′E). After sampling, soil was homogenized and sieved through a 10 mm mesh to remove coarse fragments and plant material. The homogenized soil had a pH of 7.8 and a moisture content of 14.7% (w/w) [39].

In autumn 2008, litter was collected from the Gelderse Poort region by selecting senesced leaves from standing plants [74]. Litter was collected from at least 10 individuals per plant species at multiple locations within the Gelderse Poort region. Litter was air-dried, stored in paper bags until use, chopped into 0.5×0.5 cm pieces and mixed for subsequent use in the experiment. Initial chemical composition of litter was determined on dried (at 70°C) and then ground litter (see Table 1). Total carbon (C) and nitrogen (N) were determined using a NC analyzer (Thermo flash EA 1112). Lignin content was determined according to Poorter and Villar [75]. Briefly, the litter material was subjected to polar, non-polar and acid extraction steps. The mass of the remaining residue was corrected for ash and the ash-adjusted C and N content of the residue was used to calculate lignin concentrations. This lignin fraction has been used successfully as litter quality index, but may contain small amounts of other recalcitrant C compounds besides lignin [29].

Seeds were collected in autumn 2008. Root fragments were collected for *Rorippa-*pair in spring 2009. Root fragments and seeds were surface-sterilised in a 0.5% sodium hypochlorite solution to kill potential root and seed pathogens. Root fragments of *R. sylvestris* were also rinsed with 70% ethanol, because a pilot showed higher root sprouting.

### Experiment 1: litter effects on soil respiration

In order to determine the effects of litter on soil respiration, each litter was mixed with field soil and placed in flasks. Per plant species, six flasks of 315 ml were used (four flasks for *R. austriaca* due to limited amount of available litter). Each flask received an amount of field-moist soil equivalent to 40 gram dry weight and on top of this soil a 29.6 gram mixture of soil and litter (71.6∶1) was placed, representing an average yearly amount of litter per unit of soil in temperate systems [76]. Six flasks without litter in the top layer were included as control. Soil was kept at 50% water holding capacity (WHC), which equals 17.7% w/w. Flasks were closed with a rubber septum, placed in randomized order in an incubation chamber and incubated at 10°C, which is the yearly average temperature of the Netherlands (www.knmi.nl). At days 3, 7, 15, 22 and 29, gas samples were collected from the headspace using a gastight syringe and stored in an Exetainer® vial until analysis. After each sampling, flasks were opened to allow ventilation for an hour to prevent high CO_2_ levels in the flasks and to adjust the moisture if needed by adding demineralized water. CO_2_-concentrations were measured against a reference line on a Thermo FOCUS GC equipped with a RT-QPLOT column from Restek (30 m long and 0.53 mm diameter). The average CO_2_ concentration in control pots was subtracted from the CO_2_ concentration in the pots that contained litter. Cumulative CO_2_ production was calculated for each litter type.

### Experiment 2: litter effects on soil N, enzyme activities and fungal biomass

In order to determine how litter influenced soil N availability, enzyme activities and fungal biomass, litter of each plant species was mixed with field soil and placed in cubic microcosms of 0.5 L with a surface area of 81 cm^2^. There were 15 replicates for each litter (8 replicates for *R. austriaca* and 10 for *R. sylvestris* due to limited availability of litter). Each microcosm received an amount of field-moist soil equivalent to 450 gram dry soil and on top of this soil 83 gram of the same litter-soil mixture as used in experiment 1 was added. The microcosms were incubated in a climate room at 10°C, 83% humidity and soil was kept at 50% WHC ( = 17.7% w/w). Five random microcosms were harvested after 2, 9 and 18 weeks of incubation, after which the top layer of soil was analyzed.

Available mineral N was extracted by shaking moist soil (equivalent to 10 g dry weight) in 50 ml 1 M KCl for 2 h. N-NH_4_^+^ and N-NO_3_^−^ concentrations were measured on a Technicon TrAAcs 800 auto-analyzer. pH_water_ was measured in a 1∶ 2.5 soil to water ratio. Ergosterol, a specific fungal biomarker in the cell wall, was used to measure fungal biomass. This biomarker is not present in arbuscular mycorrhizal fungi (AMF) [77]. Ergosterol was extracted from soil using an alkaline-extraction method and measured on a Dionex HPLC equipped with a C 18 reverse-phase column and a UV-detector set at 282 nm [78]. Lignin degrading enzyme activity (Mn-peroxidase) and cellulose degrading enzyme activity (endo-1,4-β-glucanase) were measured according to Van der Wal et al. [79], modified by extracting 6 gram of soil with 9 ml of milli-q water. Endo-1,4-β-glucanase is an indicator of cellulase activity and is therefore called cellulase in the main text.

### Experiment 3: litter effects on seedling germination and plant biomass production

In order to determine how litter influenced seedling germination and plant biomass production, seeds of exotic and native plant species were placed on soil that had been incubated with their own litter, as well as on soil that had been incubated with the litter of the congener. We created a series of 10 microcosms (8 for *R. austriaca*) per litter origin, which were pre-incubated for 18 weeks as in experiment 2 in order to mimic litter decomposition in winter prior to plant growth in spring. For *Artemisia* and *Senecio*, 50 seeds of exotic or native plant species were placed on half of the microcosm within the genera to create five microcosms per litter origin for each plant origin within genera. For *Rorippa*, 10 root fragments of exotic or native species were placed in the soil of half of the microcosm. Germination or sprouting rates were registered after 17 days for *Senecio*, after 22 days for *Rorippa*, and after 36 days for *Artemisia*, because the time of germination or sprouting differed between genera. After germination, seedlings or cuttings were thinned so that one seedling with median length was left. Microcosms were harvested after 9.5 weeks of incubation. All harvested plants were dried to constant weight at 70°C and weighed. Microcosms were placed in a climate chamber at 19°C/10°C and 83% humidity (average May–September growing conditions for plant species in the Netherlands, www.knmi.nl) with daylight for 16 h per 24 h.

### Data analysis

The results were analyzed with Statistica version 9.0 (StatSoft, Inc. (2009), Tulsa, USA) by considering the three genera separately. Repeated measures ANOVAs were performed per genus-pair for soil respiration with origin (litter from exotic or native plant species) as the between-subject factor. As the sphericity assumption was violated for all genus-pairs, Greenhouse-Geisser adjusted P values and degrees of freedom were calculated (Table 2). An ANOVA was performed for the effects of litter on soil per genus-pair with origin (litter from exotic or native plant species) and time (2, 9 and 18 weeks of incubation) as fixed factors. Cellulase was log-transformed to meet assumptions of ANOVA. Inorganic N concentration was log-transformed for the genera *Artemisia* and *Rorippa* and fourth-root transformed for *Senecio* to meet assumptions of ANOVA. Effects of litter origin on germination rates and plant biomass production were analyzed per genus-pair by ANOVA with litter (litter from exotic or native plant species) and plant (exotic or native plant species) as fixed factors. Germination rates were arcsine transformed and biomass was log transformed to meet assumptions of ANOVA.

## Supporting Information

Figure S1 **Effects of litter on Mn-peroxidase activity and pH.** Mn-peroxidase activity (A, B, C) and pH (D, E, F) in soil incubated with litter from exotic plant species (filled circles) or with litter from native plant species (open circles). Means (± SE) are presented for *Artemisia* (A, D), *Rorippa* (B, E) and *Senecio* (C, F).
(PDF)
