# Supplementary material for: Reciprocal Effects of Litter from Exotic and Congeneric Native Plant Species via Soil Nutrients
Source: PLoS One. 2012 Feb 16;7(2):e31596. doi: 10.1371/journal.pone.0031596 (PMC3281088; doi:10.1371/journal.pone.0031596)
Supplement: Figure S1 — Effects of litter on Mn-peroxidase activity and pH. Mn-peroxidase activity (A, B, C) and pH (D, E, F) in soil incubated with litter from exotic plant species (filled circles) or with litter from native plant species (open circles). Means (± SE) are presented for Artemisia (A, D), Rorippa (B, E) and Senecio (C, F). (PDF) [file pone.0031596.s001.pdf]

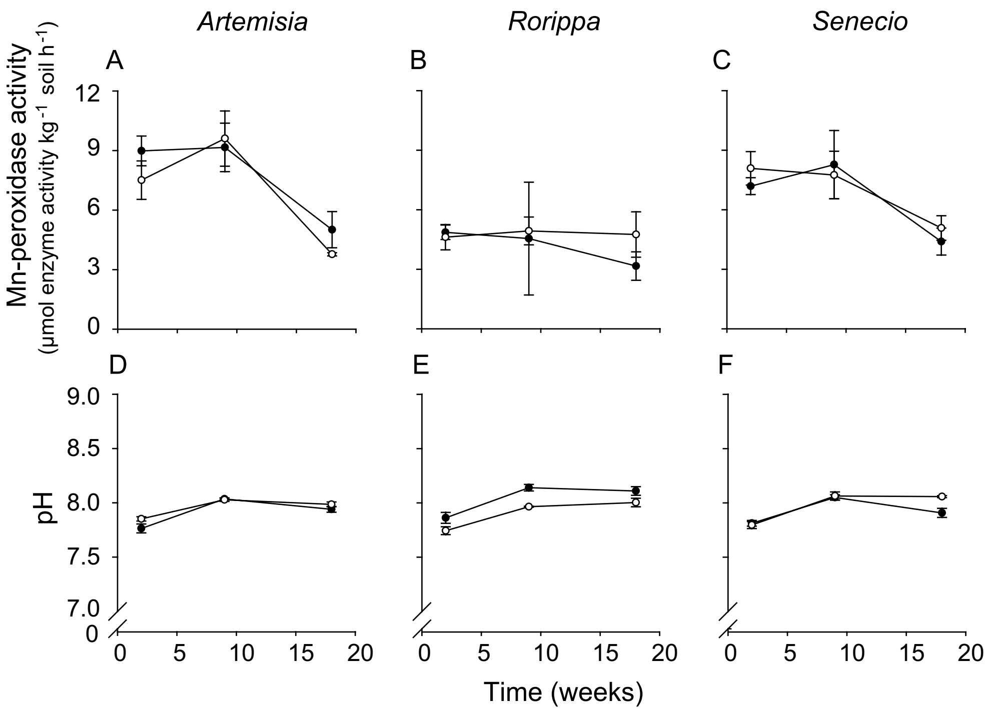

Figure S1. Effects of litter on Mn-peroxidase activity and pH. Mn-peroxidase activity (a, b, c) and pH (d, e, f) in soil incubated with litter from exotic plant species (filled circles) or with litter from native plant species (open circles). Means (± SE) are presented for Artemisia (a, d), Rorippa (b, e) and Senecio (c, f).
